# Lead Extraction in Children and Young Adults: When is the Best Time for Lead/System Replacement?

**DOI:** 10.1007/s00246-023-03320-9

**Published:** 2023-10-28

**Authors:** Andrzej Kutarski, Maria Miszczak-Knecht, Monika Brzezinska, Mariusz Birbach, Wojciech Lipiński, Wojciech Jacheć, Bettina Ziaja, Anna Polewczyk, Łukasz Tułecki, Marek Czajkowski, Dorota Nowosielecka, Katarzyna Bieganowska

**Affiliations:** 1 Department of Cardiology, Medical University of Lublin, Lublin, Poland; 2 Department of Cardiology, Children’s Memorial Health Institute, Warsaw, Poland; 3 Department of Cardiac Surgery, Children’s Memorial Health Institute, Warsaw, Poland; 4 2nd Department of Cardiology, Faculty of Medical Sciences in Zabrze, Medical University of Silesia, Katowice, Poland; 5 Department of Cardiology, Specialist Hospital in Zabrze, Zabrze, Poland; 6 Department of Medicine and Health Sciences, The Jan Kochanowski University, Kielce, Poland; 7 Department of Cardiac Surgery, Pope John Paul II Province Hospital, Zamość, Poland; 8 Department of Cardiac Surgery, Medical University of Lublin, Lublin, Poland; 9 Department of Cardiology, Pope John Paul II Province Hospital, Aleje Jana Pawła II 10, 22-400 Zamość, Poland

**Keywords:** Lead extraction in children, Lead extraction in juveniles, Complications of pacing, Lead extraction safety, Mechanical dilators

## Abstract

The best strategy for lead management in children is a matter of debate, and our experiences are limited. This is a retrospective single-center study comparing difficulties and outcomes of transvenous lead extraction (TLE) implanted ich childhood and at age < 19 years (childhood-implanted-childhood-extracted, CICE) and at age < 19 (childhood-implanted-adulthood-extracted, CIAE). CICE patients—71 children (mean age 15.1 years) as compared to CIAE patients (114 adults (mean age 28.61 years) were more likely to have VVI than DDD pacemakers. Differences in implant duration (7.96 vs 14.08 years) appeared to be most important, but procedure complexity and outcomes also differed between the groups. Young adults with cardiac implantable electronic device implanted in childhood had more risk factors for major complications and underwent more complex procedures compared to children. Implant duration was significantly longer in CIAE patients than in children, being the most important factor that had an impact on patient safety and procedure complexity. CIAE patients were more likely to have prolonged operative duration and more complex procedures due to technical problems, and they were 2–3 times more likely to require second-line or advanced tools compared to children, but the rates of clinical and procedural success were comparable in both groups. The difference between the incidence of major complications between CICE and CIAE patients is very clear (MC 2.9 vs 7.0%, hemopericardium 1.4 vs 5.3% etc.), although statistically insignificant. Delay of lead extraction to adulthood seems to be a riskier option than planned TLE in children before growing up.

## Introduction

The number of new cardiac implantable electronic device (CIED) implantations in children is not rising. However, due to advances in cardiovascular techniques, the number of young adults with repaired congenital heart as well as children with implanted cardiac devices can be expected to have increased longevity into adulthood (negligible mortality).

**Fig. 1:**
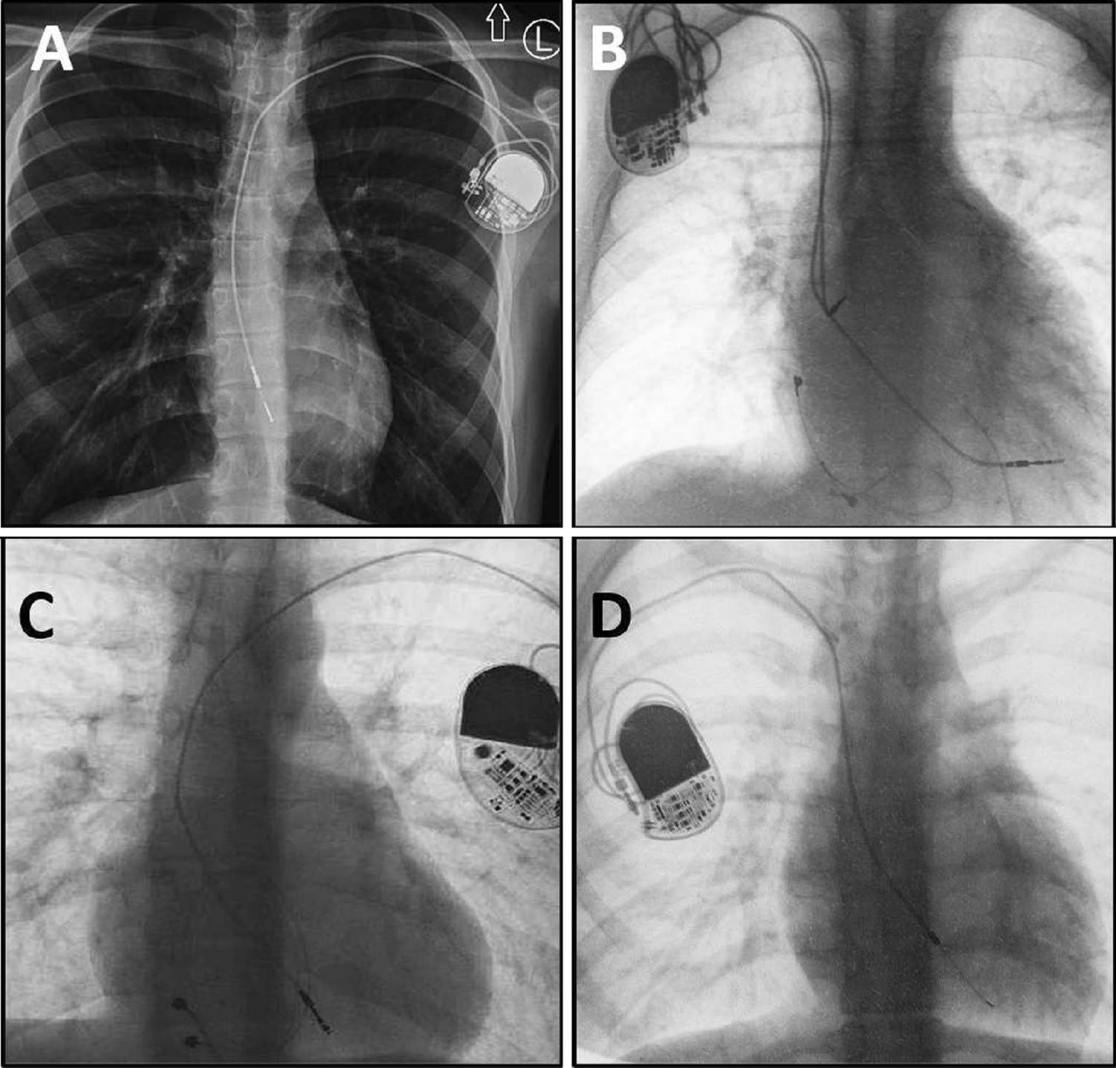
The strain of the leads caused by the growth of the body. The strain of the leads caused by the growth of the body, which is one of the causes of lead dysfunction (increase of the stimulation threshold, decrease of resistance due to damage to external insulation). Pictures typical of young adolescents with leads implanted in childhood (**A**–**D**). Abandoned epicardial leads implanted earlier in early childhood are common (**B** and **C**). In children and youth, single-chamber systems (**A**, **C** and **D**) predominate

Although reports show long-term durability of leads pacemakers (PM) 20 years, implantable cardioverter-defibrillator (ICD) 10 years [1, 2] these values are usually lower in children [3–7]. In children, mechanical damage and other types of lead dysfunction resulting from body growth, faster scarring and calcification, lead insulation breaches and a more active lifestyle limit the durability of intracardiac leads [3–7] (Fig. 1).

Lead replacement as the optimal lead management strategy for children with lead dysfunction is recommended both in previous and recent guidelines [8–10], but execution of the strategy in pediatric electrocardiology departments may differ. Differences in transvenous lead extraction (TLE) and procedure difficulty between children and adolescents have been discussed in several reports [11–17] and multicentre studies [7, 18]. Most pediatric cardiologists prefer a passive approach, that is lead removal for class 1 and 2a indications and extraction postponement in patients with class 2b indications [5, 18–20]. Considering the optimal time for lead extraction some investigators advocate waiting until children stop growing than extracting non-functional leads during physical growth, suggesting that TLE in young adults is safer [18–20].

As a result, most children, once they turn 18, are referred to adult electrophysiologists for potential lead replacement or system upgrade. Finally, most young adults become candidates for TLE at adult centers.

No previous study has investigated the effects of postponing TLE to an undefined time as long as the leads meet the criteria of functionality (without analysis of their age and their radiological image, often suggesting impending dysfunction). Furthermore, there have been no studies that compare the effectiveness and safety of TLE in adolescents with leads implanted in childhood and TLE in children. This knowledge gap prompted us to conduct this study.

The aim of the study was to compare the complexity, effectiveness, and outcomes after TLE in children (below the age of 19 years) from pediatric wards and TLE in young adults (with leads implanted in childhood) performed at a high-volume center for adult patients. We sought to determine which strategy is better for children with borderline functional leads: to delay lead replacement as long as possible or to perform TLE in childhood even for class 2b indications.

## Methods

### Study Population

This post hoc analysis used clinical data of 3741 patients who underwent transvenous lead extraction between 2006 and 2022. The first group consisted of 71 children from 5 to 19 (mean age 15.10 ± 2.95 years) who were < 19 at first CIED implantation and transvenous lead extraction (childhood-implanted-childhood-extracted patients, CICE). The second group consisted of 114 young adults from 19 to 57 (mean age 28.61 ± 9.36 years) who were < 19 at first CIED implantation but > 19 during TLE (childhood-implanted-adulthood-extracted patients, CIAE). No other exclusion criteria were used.

### Lead Extraction Procedure

The procedures of TLE were defined according to the HRS 2009 and 2017 and EHRA 2018 guidelines [8–10] on management of lead-related complications. TLE procedures were performed in a stepwise manner using mechanical systems such as polypropylene Byrd dilator sheaths (Cook^®^ Medical, Leechburg, PA, USA), mainly via the lead implant vein. If technical difficulties arose, alternative venous approaches and/or additional tools such as Evolution (Cook^®^ Medical, USA), TightRail (Phillips®, USA), lassos, basket catheters were used. Laser cutting sheaths were not used. In both study groups lead extractions were performed by the same experienced a team. Indications for TLE and type of periprocedural complications were defined according to the HRS Expert Consensus Statement on lead extraction [8, 9].

### Definitions

Clinical success, procedural success, partial radiographic success, as well as major and minor complications of TLE were defined in accordance with recommendations for lead management [8–10]. The occurrence of permanent bodily injury, significant damage to the tricuspid valve or, for example, stroke, procedure-related death despite optimal treatment, have precluded clinical or procedural success [8–10].

The risk of major complications (MC) related to TLE (points, percentage) was assessed using the SAFeTY TLE score, an online tool available at http://alamay2.linuxpl.info/kalkulator/ [21]. The EROS score was used for prediction of significant procedural complications that required emergent surgical intervention (1–3 scale) [22]. Assessment of procedure complexity was based on the MB score showing the need for use of advanced tools to achieve TLE success (0–5 points) [23], LED index referring to lead extraction difficulty based on fluoroscopy times (0–50 points) [24], and Advanced TLE Techniques (Mazzone) score to predict the necessity of using advanced extraction techniques (0–4 points) [25].

*Procedure complexity* was expressed as procedure time, i.e., time for extraction of all leads (sheath-to-sheath time) and average time of single lead extraction (sheath-to sheath/number of extracted leads). The occurrence of unexpected procedural difficulties referred to as technical problems, i.e., the circumstances that made the procedure more difficult but without complications was another indicator of procedural complexity. Most commonly it was the need to use venous approach other than the access vein, lead-on-lead scarring, fracture of the targeted lead, Byrd dilator collapse/torsion and occlusion of the access vein [26–28].

### Lead Failure

#### Mechanical Lead Damage (Electric Failure)

Sudden increase in impedance > 1500 Ohm or high-voltage impedance > 100 Ohm; > 300 nonphysiological short interventricular-intervals (“crackles”).

#### Non-damaged Lead Dysfunction

Lead failure without mechanical damage: exit/entry block (linear increase in impedance > 1500 Ohm or high-voltage impedance > 100 Ohm or a linear decrease in sensing or increase of pacing threshold to an inacceptable level level), tip dislodgement or extracardiac pacing).

### Abnormal Lead Route

#### Strain of the Leads

Is a radiological image of an electrode that has become too short over time, which may hide a properly functioning electrode or any of the above-mentioned abnormalities. Strain of the leads indicates increased difficulty in removing the lead (close contact of the lead with the venous system and cardiac structures) and indicates an increased risk of future damage to the strained lead.

#### Abnormal Lead Loop in the Heart

A radiological image in which a lead that is too long (at the time of implantation) creates an intentional or unintentional loop in the heart, atrium or ventricle, or the loop crosses the tricuspid valve. The presence of loops increases the contact of the lead with cardiac structures and scarring at the contact sites, hinders lead dilatation and increases the risk of damage to cardiac structures (including the tricuspid valve).

### Extracted Lead Designs

In our material, all implanted PM leads had a conventional design, most of them with active fixation and steroid-eluting. The ICD leads were single-coil. Active leads were most often used, also due to their isodiametric nature. VDD leads were not used in children due to their design (distance between tip and atrial ring electrodes). The studied groups did not include Medtronic 3830 lm-less leads.

### Statistical Analysis

Depending on whether the data is normally distributed continuous variables are summarized with a mean ± standard deviation (SD) or median and interquartile range (IQR). Categorical variables are summarized using counts and percentages. The significance of differences between groups was determined using the Chi^2^ test with Yates correction or Student’s *t*-test or Mann–Whitney *U* test, as appropriate. Statistical analysis was performed with Statistica 13.3 (TIBCO Software Inc.).

All patients gave their informed written consent to undergo TLE and use anonymous data from their medical records, approved by the Bioethics Committee.

## Results

Comparison of patient characteristics (Table 1) shows that age difference on the day of TLE was 13.51 years (15.10 vs 28.61 years), and age difference on the day of lead implantation 6.25 years (7.13 vs 13.38 years) and this resulted from the assumptions of the study. There was a smaller proportion of women in the CICE group than in the CIAE group (28.17 vs 44.74%). The most common indication for TLE in both groups was mechanical lead damage (electrical failure), more frequent in children (67.61 vs 43.86%).

**Table 1:** Patient characteristics, indications, and the main goal of transvenous lead extraction

	Implantation and TLE < 19 years of age	Implantation < 19 andTLE > 19 years of age	Chi^2^ test, Student’s *t*-test,
	CICE; *n* = 71	CIAE; *n* = 114	
	Mean ± SD *n* (%)	Mean ± SD *n* (%)	*P*
*Patient characteristics*			
Patient age at TLE (years)	15.10 ± 2.95	28.61 ± 9.36	< 0.001
Patient age at first CIED implantation (years)	7.13 ± 3.90	13.38 ± 4.46	< 0.001
Female	20 (28.17)	51 (44.74)	0.036
Etiology: congenital, channelopathies, neurocardiogenic, post-cardiac surgery	70 (98.57)	107 (93.86)	0.874
LVEF average (%)	64.17 ± 8.56	60.14 ± 8.83	0.002
Charlson comorbidity index (points)	0.00 ± 0.00	0.254 ± 1.08	0.016
Previous sternotomy	24 (33.80)	20 (17.54)	0.180
Valvular implant presence	1(1.4)	5 (4.39)	0.493
Indications for TLE			
Systemic infection	4 (5.63)	12 (10.53)	0.378
Local (pocket) infection	1 (1.41)	6 (5.26)	0.347
Mechanical lead damage (electrical failure)	48 (67.61)	50 (43.86)	< 0.001
Lead dysfunction (exit/entry block, dislodgement, extracardiac pacing)	8 (11.27)	13 (11.40)	0.834
Upgrade or other non-infectious indication	10 (14.09)	33 (28.95)	0.032

*CICE* childhood-implanted-childhood-extracted, *CIAE* childhood-implanted-adulthood-extracted, *SD* standard deviation, *TLE* transvenous lead extraction, *CIED* cardiac implantable electronic device, *LVEF* left ventricular ejection fraction. Valvular implants: mechanical mitral or aortic valve, biological mitral or pulmonary valve, mitral or tricuspid ring, Charlson comorbidity index: Charlson ME, Pompei P, Ales KL, MacKenzie CR. A new method of classifying prognostic comorbidity in longitudinal studies: development and validation. J Chronic Dis.1987;40:373–783

**Fig. 2:**
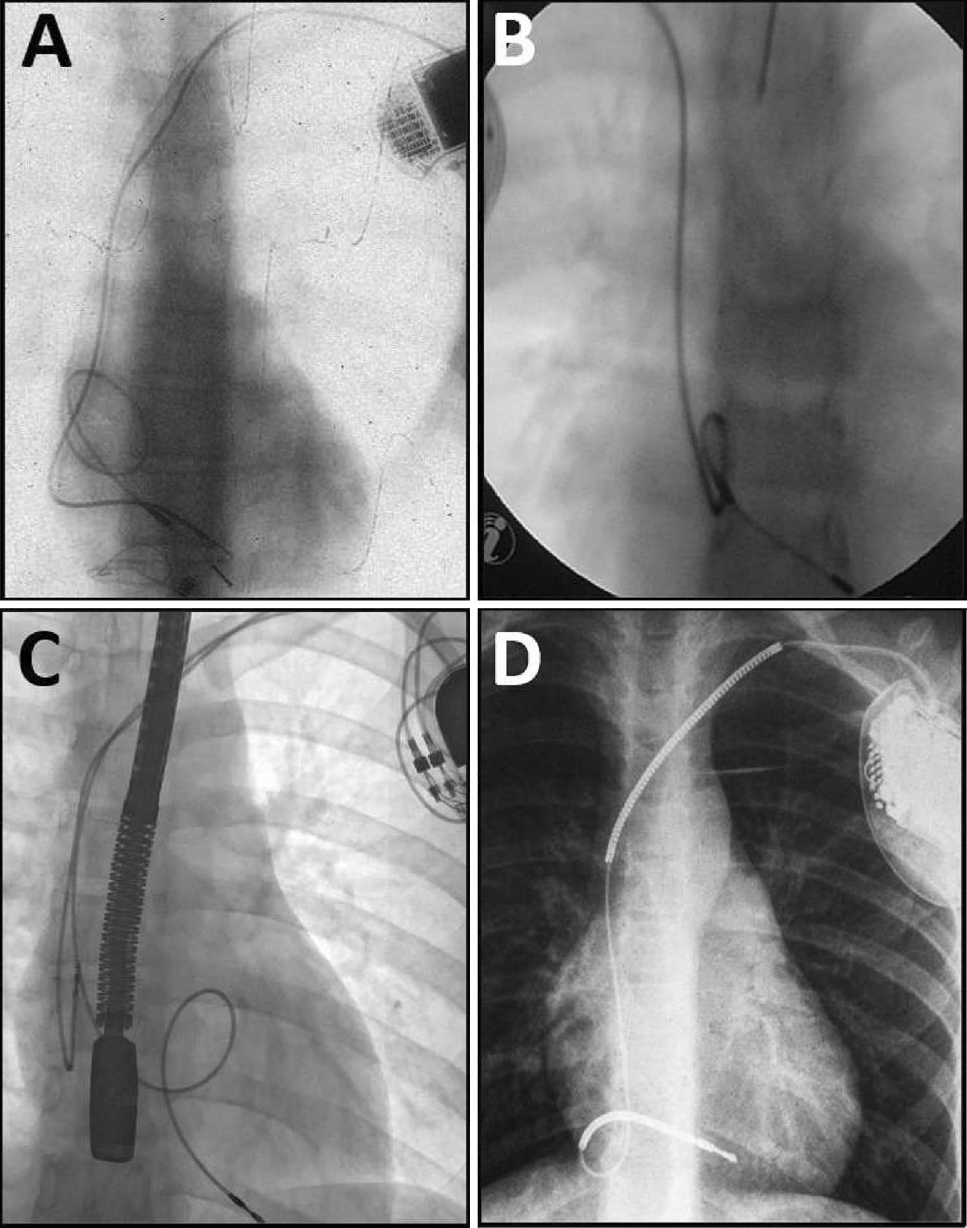
Planned lead loops to prevent progressive lead length deficit as the child grows. Planned lead loops to prevent progressive lead length deficit as the child grows (**A**–**D**). Long-term observations showed the ineffectiveness of this technique because the loops grew attached to the walls of the heart, which made it impossible to straighten them and sometimes did not prevent the dysfunction (**A**), and significantly hinders their transvenous extraction. The technique is abandoned today, however, the phenomenon is often encountered in young patients

CICE patients more frequently received simple single-chamber PM (67.61 vs 30.70%), whereas CIAE patients were more likely to receive dual-chamber PM (61.40 vs 19.72%). Furthermore, CIAE patients had more leads in the heart (1.91 vs 1.34), more frequently abandoned leads (17.54 vs 7.04%), leads on both sides of the chest (8.77 vs 2.82%) and abnormally long lead loops in the heart before TLE (Fig. 2).

A huge difference in dwell time of the oldest lead per patient before TLE (169.0 vs 95.52 months) and global implant duration (25.00 vs 9.50 years) appeared to be the most important factors influencing procedure difficulty, complexity and safety (Table 2).

**Table 2:** System and pre-procedural data

	Implantation and TLE < 19 years of age	Implantation < 19 and TLE > 19 years of age	Chi^2^ test, Student’s *t* test, Mann–Whitney *U* test
	CICE; *n* = 71	CIAE; *n* = 114	
	Mean ± SD median [IQR] *n* (%)	Mean ± SD median [IQR] *n* (%)	*P*
*System and history of pacing*			
Pacemaker: single chamber (AAI, VVI, VDD)	48 (67.61)	35 (30.70)	< 0.001
Pacemaker: dual chamber (DDD)	14 (19.72)	70 (61.40)	< 0.001
ICD-VVI	4 (6.64)	3 (2.63)	0.519
ICD-DDD	5 (7.04)	6 (5.26)	0.859
CRT-D	0 (0.00)	0 (0.00)	–
Presence of abandoned leads before TLE	5 (7.04)	20 (17.54)	0.070
Number of leads in the heart before TLE	1.34 ± 0.58	1.91 ± 0.72	< 0.001
Leads on both sides of the chest before TLE	2 (2.82)	10 (8.77)	0.042
Large lead loop in the heart before TLE	0 (0.00)	19 (16.67)	0.001
Number of CIED-related procedures before lead extraction	1.69 ± 0.802	2.35 ± 1.30	< 0.001
Dwell time of oldest lead per patient before TLE (months)	95.52 [72.96]	169.0 [119.0]	< 0.001
Global implant duration (per patient) before TLE (months	114.0 [86.64]	300.0 [219.0]	< 0.001

*TLE* transvenous lead extraction, *CICE* childhood-implanted-childhood-extracted, *CIAE* childhood-implanted-adulthood-extracted, *SD* standard deviation, *IQR* interquartile range, *AAI* single-chamber pacemaker with lead in right atrium, *VVI* single-chamber pacemaker with lead in right ventricle, *VDD* atrial sensing, ventricular sensing/pacing single-chamber pacemaker with lead in right ventricle, *DDD* dual-chamber pacemaker, *ICD-VVI* single-chamber cardioverter-defibrillator, *ICD-DDD* dual-chamber defibrillator, *CRTD* cardiac implantable cardioverter-defibrillator

Several factors allow us to predict procedural risk and difficulty, whereas retrospective analysis may shed some light on the development of major complications and procedure complexity (Table 3).

**Table 3:** Potential risk factors for major complications and procedure complexity

	Implantation and TLE < 19 years of age	Implantation < 19 and TLE > 19 years of age	Chi^2^ test Student’s *t* test Mann–Whitney *U* test
	CICE; *n* = 71	CIAE; *n* = 114	
	Mean ± SD median [IQR] *n* (%)	Mean ± SD median [IQR] *n* (%)	*P*
Number of extracted leads per patient	1.16 ± 0.44	1.75 ± 0.68	0.013
Approach other than lead access vein	2 (2.82)	11 (9.65)	0.141
Extraction of leads with too long loops in the heart	0 (0.00)	30 (26.32)	< 0.001
Extraction of abandoned lead(s) (any)	3 (4.23)	19 (16.67)	0.047
Defibrillating lead extracted	9 (12.68)	9 (7.90)	0.417
Oldest extracted lead (months)	86.52 [66.00]	169.0 [237.0]	< 0.001
Cumulative dwell time of extracted leads (months)	103.0 [76.56]	248.0 [181.0]	< 0.001
*Calculators, scales, and scores for prediction of procedure safety and difficulty*			
SAFeTY TLE score for prediction of major complications [points]	5.83 [1.38]	11.00 [6.87]	< 0.001
SAFeTY TLE score for prediction of major complications [%]	0.78 [0.28]	3.22 [5.60]	< 0.001
EROS score: 2 points	67 (94.37)	37 (32.46)	< 0.001
EROS score: 3 points	3 (4.23)	44 (38.60)	< 0.001
LED index scale [points]	9.00 [6.00]	15.00 [10.00]	< 0.001
MB score [points]	2.00 [1.00]	4.0 [1.0]	< 0.001
Advanced TLE (Mazzone) score [points]	2 [0]	3.00 [1.00]	< 0.001

*TLE* transvenous lead extraction, *CICE* childhood-implanted-childhood-extracted, *CIAE* childhood-implanted-adulthood-extracted, *SD* standard deviation, *IQR* interquartile range, *MC* major complications, *EROS* ELECTRa Registry Outcome Score,

The CIAE group was characterized by more extracted leads per patient (1.75 vs 1.16), extraction of multiple leads (three or more) (7.90 vs 1.41%), need to use other than lead vein approach (9.65 vs 2.82%), extraction of leads with too long loops in the heart (26.32 vs 0.00%), and extraction of abandoned lead(s) (16.67 vs 4.23%). Some differences were not significant, but there was a noticeable tendency (Fig. 2).

Dwell time of the oldest extracted lead (169.0 vs 86.52) and cumulative dwell time of extracted leads, i.e., the sum of extracted lead dwell times (20.67 vs 8.58 years) provide the most important information.

The bottom row of the Table 3 summarizes the usefulness of the most popular calculators, scales, and scores for assessment of procedure safety and difficulty (complexity).

The SAFeTY TLE score assessing the risk of major complications [21] showed 4.13 times higher risk of MC, the EROS 3 score [22] showed 9.13 times higher of risk of significant procedural complications that required urgent surgical intervention, the MB score 4 and 5 [23] showed 18.35 times higher need for advanced tools to achieve TLE success, the LED index exceeding 14 points [24] indicated 6.64 times higher risk of difficult TLE based on fluoroscopy time, Advanced TLE Techniques (Mazzone) [25] score 3 and 4 predicted 3.59 times higher need for advanced extraction techniques in CIAE patients as compared to CICE patients. All calculators, scales, and scores predicted a higher risk of MC and procedure difficulty (complexity) in CIAE patients.

The venue of TLE in CIAE patients has evolved with years from an EP-LAB via a cardiac surgical operating theater to a hybrid room. Lead extractions in CICE patients were performed in children’s hospital, first in the cardiac surgical operating room or later in the hybrid room. Most TLEs were done in patients under general anesthesia. Continuous TEE monitoring was introduced 7 years ago in adult departments and 4 years ago in children’s departments (Table 4).

**Table 4:** Lead extraction safety, procedure course, complexity and technical problems

	Implantation and TLE < 19 years of age	Implantation < 19 and TLE > 19 years of age	Chi^2^ test Student’s *t* test Mann–Whitney *U* Test
	CICE; *n* = 71	CIAE; *n* = 114	
	Mean ± SD median [IQR] *n* (%)	Mean ± SDmedian [IQR] *n* (%)	*P*
*Venue*			
Venue of TLE: cardiac surgical operating theater or hybrid room	69 (97.18)	72 (63.16)	< 0.001
Cardiac surgeon as co-operator	68 (95.78)	67 (58.77)	< 0.001
General anesthesia	69 (97.18)	92 (80.70)	0.003
Mandatory TEE for monitoring lead extraction	29 (40.85)	54 (47.37)	0.476
*Procedure difficulty and complexity*			
Procedure duration (sheath-to-sheath) [minutes]	9.00 [12.00]	19.00 [24.00]	< 0.001
Average time of single lead extraction (sheath-to-sheath/number of extracted leads) [minutes]	9.00 [14.00]	11.50 [18.00]	0.247
Non-functional superfluous lead extracted	3 (4.23)	19 (16.67)	0.021
Unexpected technical problems during TLE (any)	22 (30.99)	49 (42.98)	0.134
Occlusion of the access vein (subclavian region)	15 (21.13)	20 (17.54)	0.680
Lead-on-lead scarring (intraoperative diagnosis)	0 (0.00)	17 (14.91)	0.005
Byrd dilator collapse/torsion/”fracture”	6 (8.45)	17 (14.91)	0.286
Lead break during extraction	6 (8.45)	17 (14.91)	0.286
Need to change venous approach	1 (1.41)	11 (9.65)	0.057
Number of unexpected procedural difficulties	1.27 ± 0.46	1.74 ± 1.02	0.003
Multiple (> 1) unexpected procedural difficulties	6 (8.45)	24 (21.05)	0.040
Use of additional tools			
Evolution (old and new) or TightRail	2 (2.82)	10 (8.77)	0.196
Metal sheaths	15 (31.13)	21 (18.42)	0.794
Lasso catheters/snares/basket catheters	8 (11.27)	19 (16.67)	0.425

*TLE* transvenous lead extraction, *CICE* childhood-implanted-childhood-extracted, *CIAE* childhood-implanted-adulthood-extracted, *SD* standard deviation, *IQR* interquartile range, *TEE* transoesophageal echocardiography

Procedure duration expressed as a median of sheath-to-sheath time (19.00 vs 9.00 min) was longer in CIAE patients, which was a result of a larger number of extracted leads; the median of single lead extraction time (sheath-to-sheath/number of extracted leads) was not significantly different from that in CICE patients (11.50 vs 9.00; *P* = 0.247).

Non-functional superfluous leads were more commonly extracted in CIAE patients (16.67 vs 4.23%). It is also worth underlining that young adults were 4 times more likely to undergo extraction of abandoned leads (which is a risk factor for major complications of TLE) compared to children.

Extraction procedures can be prolonged or complicated by occlusion of the access vein (subclavian region), Byrd dilator collapse/torsion/“fracture,” lead break during extraction, lead-on-lead scarring, need to use alternative venous approach, loss of free lead portion, need to extract non-targeted leads. The problems must be solved, thus prolonging the procedure (they are not major or minor complications of TLE). As Table 4 shows, unexpected technical problems occurred twice (or more) as frequently in CIAE patients. It should be highlighted that multiple (in the same patient) unexpected difficulties were nearly 3 times more common in CIAE patients. In CIAE patients the second-line tools (Evolution, TightRail, metal sheaths, lasso catheters / snares) were used 1.5 (or more) times more often than in children.

Major complications were more often in CIAE than CICE patients (7.02 vs 2.87%), i.e., hemopericardium (5.26 vs 1.41%), need for urgent cardiac repair (5.26 vs 1.41%). (Table 5).

**Table 5:** Procedure efficacy, complications, and postoperative outcomes

	Implantation and TLE < 19 years of age	Implantation < 19 and TLE > 19 years of age	Chi^2^ test
	CICE; *n* = 71	CIAE; *n* = 114	
	*n* (%)	*n* (%)	*P*
Major complications (any)	2 (2.87)	8 (7.02)	0.371
Hemopericardium	1 (1.41)	6 (5.26)	0.347
Tricuspid valve damage during TLE (severe)	1 (1.41)	2 (1.75)	0.423
Rescue cardiac surgery	1 (1.41)	6 (5.26)	0.347
Death: procedure-related (intra-, post-procedural)	0 (0.00)	0 (0.00)	
Death: indication-related (intra-, post-procedural)	0 (0.00)	0 (0.00)	
Partial radiographic success (remained tip or < 4 cm lead fragment)	9 (12.68)	19 (16.67)	0.599
Complete clinical success	69 (97.18)	109 (95.61)	0.124
Complete procedural success	60 (84.51)	96 (84.21)	0.984
No permanently disabling complication or death	1 (1.41)	2 (1.75)	0.423

*TLE* transvenous lead extraction, *CICE* childhood-implanted-childhood-extracted, *CIAE* childhood-implanted-adulthood-extracted,

TLE effectiveness expressed as lack of partial radiographic success (16.67 vs 12.68%), clinical (95.61 vs 97.18%) and procedural (84.21 vs 84.51%) was lower in CIAE patients. Summing up, in young patients with leads implanted in childhood (CIAE) all complications occurred more frequently than in children: major complications (any)—2.5 times, hemopericardium—3.73 times, significant or severe tricuspid valve damage—1.2 times more frequently (Table 5).

## Discussion

This study attempts to answer the question which is better: to extract leads in childhood (for class 2b indications) during physical growth or to wait for lead replacement until children stop growing or perhaps to postpone it as far into the future as possible. Our analysis showed that in young adults with leads implanted in childhood (CIAE) implant duration was 1.8 times longer than in children (CICE). As a consequence, CIAE patients underwent more complex procedures due to the occurrence of various unexpected technical problems, and they were 2–3 times more likely to require second-line or advanced tools compared to children.

Moreover, all complications occurred more frequently in CIAE than in CICE patients: major complications (any)—1.6 times, hemopericardium—2.6 times, significant and severe tricuspid valve damage—3.3 times more frequently. Finally, we demonstrated that TLE was more difficult and associated with higher risk in young adults than in children.

The PACELEAD survey on lead extraction in children and adults [18] demonstrated that > 70% of responders favored lead abandonment for class 2b indications. The survey showed a tendency toward lead abandonment over lead extraction, especially in complex cases. Lead abandonment is still an accepted strategy in many hospitals [5, 14, 18–20, 29].

Although the risk associated with TLE can be slightly higher in children [7, 11–18], there are several tools (calculators, scores, scales) that allow us to predict the real risk of TLE in individual patients [21–25]. The risk of major complications can be assessed using the SAFeTY TLE score [21] and EROS score [22], whereas procedure complexity can be predicted using the MB score, LED score, Advanced LE score [23–25]. We showed that all the calculators, scales, and scores indicated much more difficult and riskier TLE in young people with leads implanted in childhood (CIAE) than in children (CICE).

As mentioned in the Introduction section, transitioning from childhood to adulthood with old or very old leads, if still officially “functional,” poses an additional challenge. Correct or only “acceptable” values of pacing/sensing/impedance do not indicate long-term lead durability because of body growth [1, 3–6, 12, 18]. Such functional leads can be strained or coiled [30, 31], strongly adherent to the heart [11–16]. They are usually covered with a thick film of calcified scar tissue, which damages external lead insulation [32]. Limited lead durability, especially in children and young patients [3–7, 14, 18] creates the need for lead replacement or new lead implantation with abandonment of non-functional leads [18]. Only a few reports described the problems related to abandoned leads in children and young adults [6, 13, 19, 20, 29]. Our experience shows that lead abandonment approach in children, juveniles, and young adults may create much more serious problems 10–20 years later. Silvetti et al. in their study reported that two of 18 patients with abandoned leads developed lead endocarditis at 5 and 10 years after lead abandonment (11.1%) [20].

We selected eight reports on TLE in children and juveniles [7, 11–18] comparing mean patient age, implant duration, major complications, and occurrence of procedure-related death. (Table 6) There are 5 reports (151 children) in which mean patients age was < 18 years (about 12.4 years) and 2 reports (1023 children, juveniles, and young adults) in which mean patients age was > 18 years (about 19.3 years). There is no report on TLE in a specific group of adult patients with leads implanted in childhood. The Table 6 shows that mean age of children and young adults in the literature is 18.1 years and average dwell time of extracted leads is 38.8 months. The respective values in our groups were 15.10 years and 94.9 months for children (CICE), and 28.5 years and 173.7 months for adults (CIAE) (for uniformity, age of leads was expressed as a mean). This indicates that adults with leads implanted in childhood (CIAE) are very specific candidates for TLE. The rate of major complications reported in the literature was 3.8% in total, whereas in our study the rate of major complications was 2.8% in children (CICE group) and as high as 7.0% in adults (CIAE group).

**Table 6:** Literature analysis. Studies in children and juveniles (all available studies). Comparison of mean patient age, mean implant dwell time, major complications, procedure-related death in relationship with patient age

Ref	Year. Journal. Author	No of pts	Mean age of pts (years)	Mean lead dwell time (months)	Major complications (%)	Procedure related death (%)
[7]	2013 Atallah J Circulation (Multi-Institutional the Pediatric Lead Extractability and Survival Evaluation (PLEASE) Study)	879	18.6	28.8	4.00	0.00
[11]	1996 Friedman RA1 Pacing Clin Electrophysiol	13	13.1	54.0	0.00	0.00
[12]	2003 Cooper JM J Cardiovasc Electrophysiol	14	17.9	42.4	0.00	0.00
[13]	2006 Moak JP Pacing Clin Electrophysiol	25	10	49.4	8.00	0.00
[14]	2009 Dilber E Med Princ Pract	30	12	46.0	2.80	0.00
[15]	2010 Cecchin F Circ Arrhythm Electrophysiol	144	21.5	86.8	2.80	0.00
[16]	2010 Zartner PA Europace	22	12.9	61.2	0.00	0.00
[18]	2014 McCanta AC Pacing Clin Electrophysiol	47	15.0	68.4	4.80	0.00
Studies in children and juveniles. Summary		1174	18.14	38.82	3.78	0.00
Our group of children (CICE)		71	15.1	94.9	2.87	0.00
Our group of adult patients with lead implanted in childhood (CIAE)		114	28.5	173.7	7.02	0.00
CICE + CIAE (weighted average)		185	23.36	143.5	5.43	0.00

*CICE* childhood-implanted-childhood-extracted, *CIAE* childhood-implanted-adulthood-extracted

The conclusion from this Table 6 is that a passive approach to lead management and delay of lead replacement until adulthood, or occurrence of serious complications in children creates a group of patients in whom lead extraction is most difficult and risky.

Our results further support the attitudes of Cecchin et al. who clearly explained that “Our approach has been to embrace the logic that most individuals with expected longevity > 20 years should have nonfunctioning leads removed to leave room for the future and avoid a more difficult procedure down the line” [15].

“Prophylactic extraction” of functional but old or very old leads is still a matter of debate. Evidence collected over the last 3 decades shows that the risk associated with lead extraction doubles every 3 years and lead lifetime is limited [1–7, 14, 18, 29]. An important issue which remains to be solved is to decide which is better: prophylactic lead replacement in children who stopped growing or waiting for dysfunction of gradually older leads. Our experience shows that symptomatic patients with unexpected lead failure are at risk of being admitted to a provincial hospital where they receive an additional lead without even considering lead replacement. It is the most common reason for lead abandonment which translated into more complex lead extraction at a later time (demonstrated in our study and previous studies in adults) [33, 34]. A general conclusion is that a passive approach to lead management and delay of lead replacement far into the future and the occurrence of serious complications in children and in juveniles creates a group of patients in whom lead extraction is most difficult and risky.

This paper provides some clues to the electrophysiologists performing TLE in adults, and gentle suggestions for future revision of the guidelines on lead management strategy in patients with leads implanted in childhood.

Although the PACES recommendations recommend adapting the implanted system optimally to the disease and type of cardiac arrhythmia, the selection of the CIED system is often determined by factors such as the expected height of the child and the expected deficit in lead length, faster lead wear and tear than in adults and the expected need to replace the lead after 10–12 years—cause the some children to receive a single-chamber system. This is not a generally accepted strategy, but it seems at least acceptable [35].

The main limitation of this study is that it involved only patients undergoing transvenous lead extraction, whereas there was no possibility of assessing patients with system upgrade without TLE procedures. The organizational model of TLE procedures evolved over time (procedures in adults 03, 2006–08, 2022, procedures in children 01, 2007–08, 2022). A mechanical-only approach to lead extraction was used throughout the study (no laser energy). Finally, all procedures in this study were performed by the same very experienced extractor and his team (co-operator, heart surgeon, nurses), therefore it may not give an overview of TLE safety and efficacy in children and young adults.

### Our Most Important Finds

Young adults with leads implanted in childhood were more likely to undergo complex procedures with major complications compared to children. Implant duration was significantly longer in young adults than in children, which appeared to be the most important factor influencing procedure safety and complexity. Compared to children, young adults had longer and more complex extraction procedures, and they were 2–3 times more likely to require second-line tools. All technical complications were also more common, but the rates of complete radiographic, clinical and procedural success were comparable.

## Conclusion

Delay of lead extraction to adulthood seems to be a riskier option than planned TLE in children before growing up.
